# New Nitro-Laterally Substituted Azomethine Derivatives; Synthesis, Mesomorphic and Computational Characterizations

**DOI:** 10.3390/molecules26071927

**Published:** 2021-03-30

**Authors:** Mohamed A. El-atawy, Magdi M. Naoum, Salma A. Al-Zahrani, Hoda A. Ahmed

**Affiliations:** 1 Chemistry Department, Faculty of Science, Alexandria University, P.O. 426 Ibrahemia, Alexandria 21321, Egypt; 2 Chemistry Department, Faculty of Science, Taibah University, Yanbu 46423, Saudi Arabia; 3 Department of Chemistry, Faculty of Science, Cairo University, Cairo 12613, Egypt; magdinaoum@yahoo.co.uk; 4 Department of Chemistry, College of Sciences, University of Ha’il, Ha’il 2440, Saudi Arabia; s.alzahrane@uoh.edu.sa

**Keywords:** lateral nitro-substituent, azomethine liquid crystals, mesophase stability, geometrical structure, DFT

## Abstract

Two new homologues series, based on two rings of the azomethine central group bearing the terminal alkoxy group of various chain lengths, were prepared. The alkoxy chain length varied between 6 and 16 carbons. The other terminal wing in the first series was the F atom, and the compound is named N-4-florobenzylidene-4-(alkoxy)benzenamine (**I***n*). The second group of compounds included a lateral NO_2_ substituent in addition to the terminal F atom, named N-(4-fluoro-3-nitrobenzylidene)-4-(alkyloxy)aniline (**II***n*). Mesomorphic and optical properties were carried out via differential scanning calorimetry (DSC) and polarized optical microscopy (POM). Elemental analyses, FT-IR, and NMR spectroscopy were carried out to elucidate the molecular structures of the synthesized groups. Mesomorphic investigations indicated that all the synthesized homologues (**I***n*) were monomorphic, possessing the smectic A (SmA) phase monotropically, while the second group (**II***n*) members were non-mesomorphic. The experimental data indicated that the formation of the mesophase is affected by the protrusion of the lateral nitro group. The disruption of the mesophase in the second group was attributed to the increase of its molecular width, which affects its lateral intermolecular interactions. The computational simulations were in agreement with the experimental data. On the other hand, the location of NO_2_ group within the molecular geometry increased the melting temperature of the molecule, and thus, affected their thermal and physical properties. By discussing the estimated parameters, it was found that the molecular architecture, the dipole moment, and the polarizability of the investigated compounds are highly affected by the electronic nature and position of the terminal and lateral substituents as well as their volumes.

## 1. Introduction

Today, liquid crystalline (LC) materials prove to have wide area of technological applications as optical displays, emitting-diodes, and photoconductors based on organic compounds [1,2,3]. Geometric characteristic relationships are important tools to synthesize a suitable structural shape to achieve the desired properties for specific industrial applications [4,5,6,7]. Molecular shape enables some considerable modifications in the mesomorphic properties and plays an important role in the formation, kind, and thermal stability of the produced mesophase [8,9,10,11,12,13,14,15]. In addition, the choices of the laterally attached groups, terminal wings, as well as the mesogenic linkages are important criteria in the formation of thermotropic LC for proper characteristic applications.

The NO_2_ group is one of the most important substituents of organic derivatives [16,17,18]. The incorporation of a nitro substituent into a molecular structure significantly increases the electronegativity of the molecule [17,18,19,20,21,22,23,24]. The strong mesomeric property of the NO_2_ moiety induces a positive charge on the N atom, and thus, increases the electronegativity of the group [17,18]. The resonance approach has been supported by dipole moment investigations [25], NMR, and the chemical electron-withdrawing character of the nitro group, which affects the measurements [26,27,28]. For the development of the mesomorphic range and mesophase stabilities of the prepared materials, an effective strategy for modification of the architectures of compounds is necessary. Therefore, the insertion of a compact lateral and terminal group into the calamitic thermotropic liquid crystals [29] is important. Generally, adjusting the type and size of terminal substituents can offer good changes in mesophase behavior [30,31]. In addition, a small change of the terminal lengths or electronic nature of groups can alter the terminal and parallel interactions [13,15].

Schiff base linkages have been broadly employed in the preparation of numerous LC derivatives [32,33,34,35,36]. Most reports have been focused on Schiff bases since the discovery of nematogenic 4-methoxybenzylidene-4′- butylaniline at room temperature [37]. Recently, several thermotropic Schiff base liquid crystals based on two rings were documented [32,38,39,40,41,42,43,44,45,46,47].

Simulations of the theoretical calculations for predicting the molecular geometry are more interesting for the estimation of the thermal properties, and we correlated them with the experimental findings data [8,9,48,49,50,51,52].

**Scheme 1:**
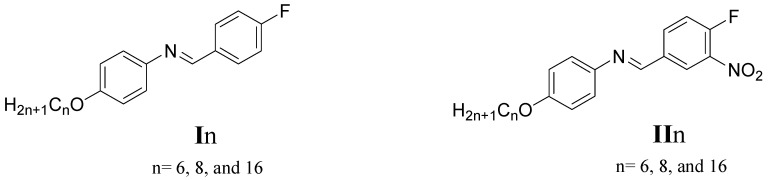
Synthesized groups **I***n* and **II***n*.

The aim of the present investigation is to synthesize new di- and tri-substituted azomethine liquid crystals based on two benzene rings (Scheme 1). Moreover, we also aimed to investigate the effect of the introduction of lateral nitro-substituents to the linear structure of a terminally substituted fluorine molecule, the effect on the mesomorphic, and the optical behavior of the synthesized architectures. Furthermore, geometrical parameters estimated from density functional theory (DFT) calculations of different sizes of attached substituents were correlated to explain the outcome of the experimental results.

## 2. Results and Discussion

### 2.1. Mesomorphic and Optical Behavior

**Figure 1:**
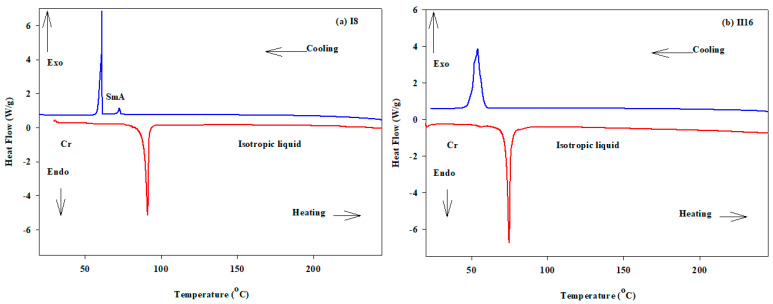
Differential scanning calorimetry (DSC) thermograms of **I***8* and **II***16* derivatives upon the second heating/cooling scan with a rate of 10 °C/min.

**Figure 2:**
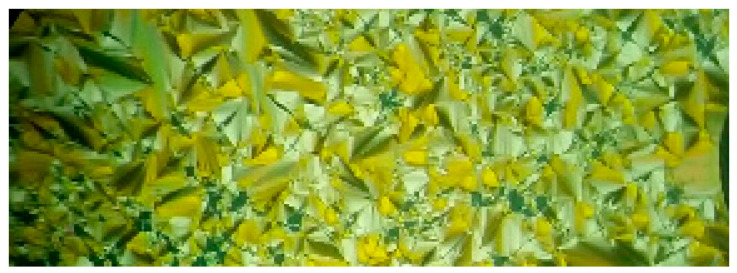
Polarized optical microscopy (POM) texture of SmA phase upon cooling of compound **I***8* at 80.0 °C, as an example.

**Figure 3:**
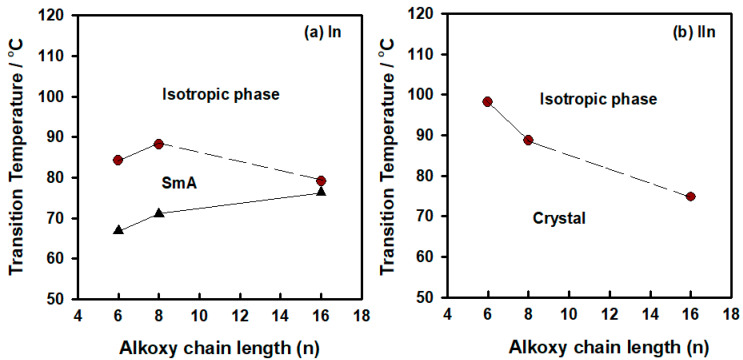
DSC transitions of synthesized groups (**a**) **I***n* and (**b**) **II***n*; the smectic A mesophase for series **I***n* was observed monotropically (upon cooling).

The transition temperatures of the synthesized groups N-4-florobenzylidene-4-(alkoxy)benzenamine (**I***n*) and N-(4-fluoro-3-nitrobenzylidene)-4-(alkyloxy)aniline (**II***n*), as well as their associated enthalpies, were derived from differential scanning calorimetry (DSC) measurements, and are summarized in Table 1. The mesophases were identified via polarized optical microscopy (POM). DSC thermograms are illustrated in Figure 1 upon the heating/cooling for the **I***8* and **II***16* derivatives, taken as examples. The POM images showed a focal-conic fan texture for the smectic A (SmA) phase for group **I***n* upon cooling (Figure 2). In order to ensure the thermal stabilities of the investigated derivatives, DSC measurements were performed for second heating scans. In addition, DSC evaluations were confirmed by the POM image observations. In order to investigate the effect of the terminal alkoxy chain groups on the mesomorphic properties of synthesized groups, the relationship of the graphical transition temperatures DSC data was illustrated and is shown in Figure 3. Members of the nitro-free, terminal fluoro-substituted homologues (group **I***n***)** exhibited only one transition peak upon heating and two transition peaks on the cooling cycles, as indicated by their DSC thermograms, which were assigned to Cr-to-isotropic liquid phase (during heating) in addition to the SmA-to-Cr phase (upon cooling). On the other hand, all compounds of di-substituted F and NO_2_ moieties exhibited only one transition peak upon the heating and cooling scans, which was assigned to the Cr-to-isotropic liquid mesophase. These data were consistent with the POM investigations. Table 1 and Figure 3 reveal that the laterally neat NO_2_ homologues, **I***n*, only possessed the smectic A phase monotropically. Moreover, there was, as usual, no regular change of melting temperatures with the increase of the terminal alkoxy chain length (*n*). It was previously reported that the position of the lateral NO_2_ group within the molecular structure also increases the melting transition temperature [28]. Their SmA stability and range also increases gradually with *n*. The homologue with the shortest alkoxy chain (**I***6*) exhibited the lowest thermal smectic stability at 66.9 °C with a smectogenic temperature range of 5.4 °C [32,53]. The higher homologue **I***16* possessed smectogenic stability at 76.3 °C and temperature range of more than 56.3 °C (see Supplementary Materials). The small size of the F atom enables its introduction into mesomorphic structures without any steric disruption, and consequently, LCs mesophases can still be observed. The high polarity also serves to enhance the optical morphology, transition phenomena, and other physical parameters. Additionally, the polar terminal F changes the polarizability and the dipole moment of the whole molecular structure to an extent, depending on its location and orientation in the molecule. This was reflected on the mesophase and optical behaviors of the investigated molecule. The insertion of lateral nitro group near the F atom in the molecule is associated with a little change in the molecular shape, but changes do exist in the polarity and orientation of the dipole moments. The mesomorphic measurements revealed that, all the homologues of group **II***n* that were laterally NO_2_-substituted proved to be non-mesomorphic. This may be attributed to the large volume of the nitro group and the high-molecular packing that not only disrupts the SmA mesophase, but also prevents the formation of any mesophase. It was documented [54] that the type of phase and its stability are mainly dependent on the dipole moment of the mesogenic part of the molecule, which varies according to the incorporated polar groups and its steric effect, which changes with the volume and location of the substituent.

### 2.2. DFT Calculations

#### 2.2.1. Geometrical Simulations and Thermal Parameters

**Figure 4:**
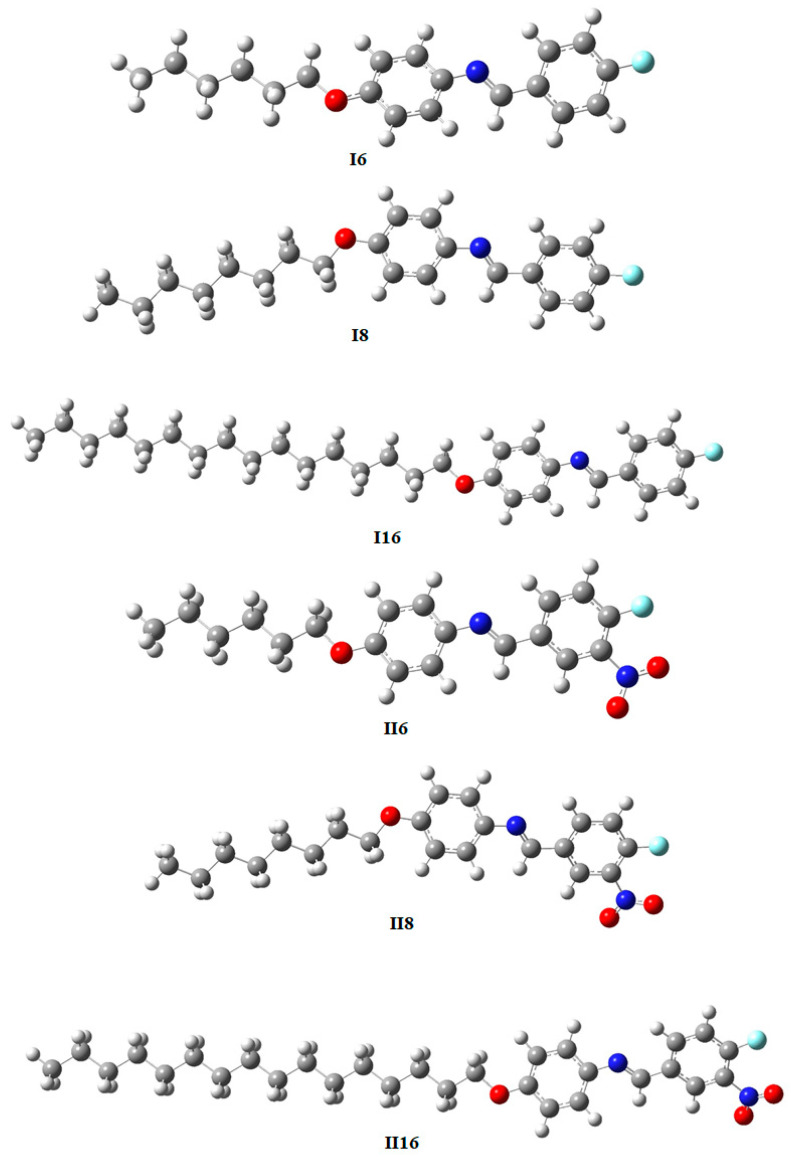
Optimized structures of molecules of the investigated homologues series **I***n* and **II***n*, calculated at the B3LYP/6-311G** level.

In order to study the effect of mono- and di-substituted compact polar groups on the mesomorphic properties of two-ring azomethine liquid crystals homologues, computational calculations for each of the investigated groups were studied. The estimations were established between the quantum chemical parameters, calculated by DFT theoretical calculations, and the experimental findings for the synthesized groups **I***n* and **II***n*. Calculations were estimated in the gas phase at the B3LYP level using 6-311G** as the basis set of predicted the optimum and stable molecular geometries. All computational calculations are performed by Gaussian 09W package [55]. The results revealed that all homologues exhibited a linear structural shape with planar geometry. In addition, the length of the terminal alkoxy chain (*n*) and the introduction of the polar NO_2_ substituent into the molecular skeleton highly affected the structural geometry of the molecule (Figure 4).

The thermal energy, enthalpy, Gibb’s free energy, and entropy, as well as the total energy of the synthesized groups at room temperature, were calculated at the B3LYP/6-311G (d, p) level and collected, as shown in Table 2. Moreover, other predicted geometrical parameter values are summarized in Table 3. The data in Table 2 and Table 3 show that the thermal and geometrical parameters, which were predicted from DFT calculations, were highly affected by the electronic nature of the lateral substituent (NO_2_). The aspect ratio, polarity, polarizability, rigidity of the cores, and the architecture shape of the individual molecules, as well as the attached polar groups, were essential parameters to affect the type and thermal stability of the observed mesophase [12], in addition to the competition between the intermolecular lateral and terminal interactions, all impacted the mesomorphic characteristics of the resulting molecules. The dipole moment of the whole molecules was highly affected by the electronic nature and position of the polar substituents (F and NO_2_). The laterally NO_2_-substituted group (**II***n*) exhibited the highest dipole moment compared to the laterally neat first group (**I***n*). Their dipole moment values changed because of the inclusion of the lateral NO_2_ group, from 3.4343 to 8.3129 Debye for **I**6 and **II***6*, respectively, from 3.2523 to 7.2100 Debye for **I***8* and **II***8*, respectively, and from 3.5361 to 8.3918 Debye for **I***16* and **II***16*, respectively. The data of the estimated dipole moment illustrate the non-mesomorphic properties of group **II***n*. The high dipole moment resulted from the highest lateral electron-withdrawing NO_2_ group, which could make a high lateral interference that opposed the arrangement of molecules, thus preventing mesophase formation. Further, the high electronegativity of the terminal F substituent enhanced the SmA-phase formation in group **I***n*. Moreover, the large volume of the NO_2_ group leads to steric hindrance, and thus, disrupts the molecular packing of molecules. In Table 3, the ionization energy and electron affinity, calculated as I.E = −E_HOMO_ and E.A = −E_LUMO_, respectively, are included [56]. Their calculated values almost were not affected by the increment of the alkoxy chain length (*n*) in either group.

The polarizability is another factor [54,57,58] that affects the phase transitions. Table 3 shows that the calculated polarizability increased as the length of terminal alkoxy chain increased for both investigated groups **I***n* and **II***n*. On the other hand, the introduction of polarizable group affects the dipole moment and the polarizability of the molecule, and accordingly enhances the intermolecular attraction between molecules and the bulk terminal group, facilitating molecular space-filling between the terminal moieties [5].

#### 2.2.2. Frontier Molecular Orbitals (FMOs)

**Figure 5:**
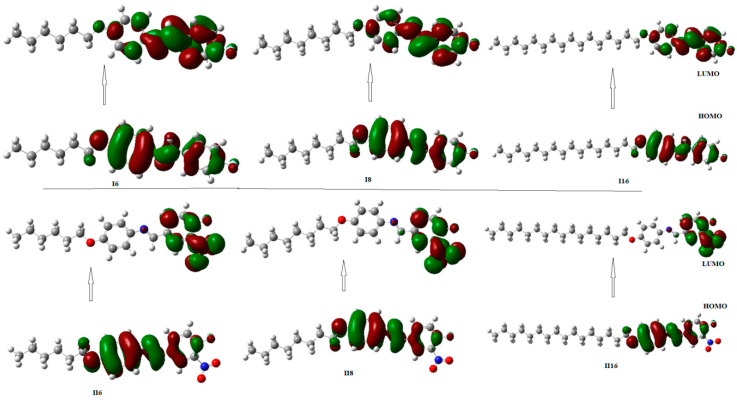
Estimated geometry for frontier molecular orbitals (FMOs) of the investigated groups, **I***n* and **II***n.*

Optical studies of non-linear optical (NLO) materials are highly affected by the energy difference between the FMOs, highest occupied molecular orbital (HOMO), and lowest unoccupied molecular orbital (LUMO) [56,59,60]. Figure 5 represents the estimated ground-state density surface plots for the FMOs of the investigated groups **I***n* and **II***n*. Table 3 shows the collected results of the FMO energy gaps. As shown in Table 3 and Figure 5, the electron densities of the sites that contributed to the formation of the HOMOs and the LUMOs were localized on each of the azomethine linkage and polar substituent F and NO_2_ groups. Moreover, the energy gap of FMOs is slightly dependent on the length of alkoxy chain (*n*). Furthermore, the mesomorphic properties of the calamitic mesogens develop the molecular–molecular interactions that essentially depend on the geometry of the compounds, the polarizability of the terminal and lateral polar substituents, as well as the stereo electronic properties of the whole molecule.

#### 2.2.3. Molecular Electrostatic Potential (MEP)

**Figure 6:**
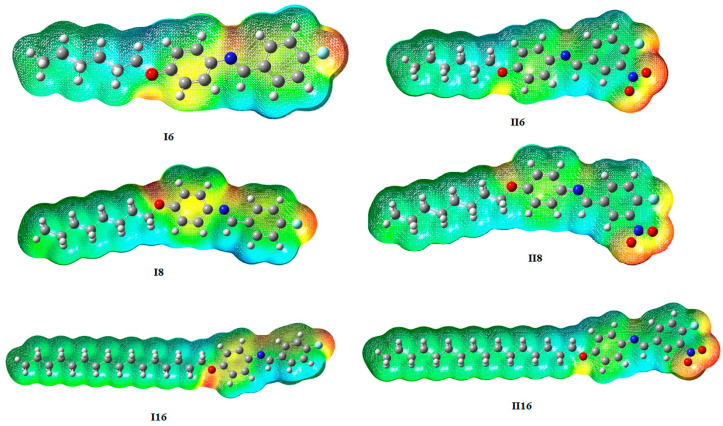
Molecular electrostatic potentials (MEP) of groups **I***n* and **II***n*.

Charge distribution maps for the present investigated series, **I***n* and **II***n*, were estimated under the same basis sets according to the molecular electrostatic potential (MEP, Figure 6). For group **I***n*, the negatively charged atomic sites (red regions) were localized on the oxygen atoms and the terminal F, as well as the nitrogen atom of the azomethine linkage. In the second group (lateral NO_2_ series), the red regions of negatively charged atomic sites were highly localized on the two oxygen of the lateral NO_2_ group, on terminal F, and on the nitrogen atom of the azomethine moiety. The alkyl chains in both groups showed the least negatively charged atomic sites (blue regions). As shown in Figure 6**,** the electronic nature of the terminal and lateral polar substituents, their size, as well as the conformation of the attached group, were more effective on the orientation of the charge distribution map; this could effect the type and thermal stability of the mesophase by altering the competitive interaction between end-to-end and side-to-side interaction. The correlation between the estimated theoretical charge distribution and the mesomorphic properties measurements was recently documented [8,12,61]. The alteration of the charge distribution on the whole molecules, because of the mesomeric interactions, enhanced the terminal aggregation to induce the N mesophase, while the parallel interaction induced the smectic A mesophase. Furthermore, the wide separation of the charges, as in case of the steric hindrance in the lateral NO_2_ group in the homologues of series **II***n*, prevented the mesophase formation, i.e., the insertion of the lateral nitro moiety disrupted the SmA phase and resulted in non-mesomorphic analogues. This can be explained in terms of the increased conjugation between the nitro group and azomethine linking moiety, which might increase the molecular non-planarity and non-linearity or attribute to the reduction of intermolecular interactions due to the broadening of the molecules.

## 3. Experimental

### 3.1. Materials

4-hexyloxyaniline, 4-(octyloxy)aniline, 4-(hexadecyloxy)aniline, 4-fluorobenzaldehyde, nitric acid, sulfuric acid and ethanol were purchased from Sigma Aldrich (Germany).

### 3.2. Synthesis

**Scheme 2:**
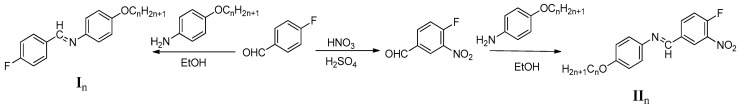
Synthesis of N-4-florobenzylidene-4-(alkoxy)benzenamine (**I***n*) and N-(4-fluoro-3-nitrobenzylidene)-4-(alkyloxy)aniline (**II***n*).

The investigated compounds **I***n* and **II***n* were synthesized according to the following scheme (Scheme 2) and see Supplementary Materials:

**N-4-fluorobenzylidene-4-(hexyloxy)benzenamine, I*6***

Yield: 97.0%; mp 84.0 °C, FT-IR (ύ, cm^−1^): 2930, 2850 (CH_2_ stretching), 1630 (C=N), 1560 (NO_2_), 1365 (NO_2_). ^1^H NMR (500 MHz, CDCl_3_) δ 10.01 (s, 1H), 8.47 (s, 1H), 7.92 (d, *J* = 8.0 Hz, 1H), 7.85 (d, J = 7.7 Hz, 1H), 7.54 (d, *J* = 7.9 Hz, 1H), 7.45 (dd, *J*^H-F^ = 12.8, *J*^H-H^= 8.4 Hz, 1H), 7.31 (d, *J* = 8.0 Hz, 1H), 7.25–7.13 (m, 1H), 7.03–6.83 (m, 1H), 4.00 (t, *J* = 6.5 Hz, 2H), 3.95 (t, *J* = 6.3 Hz, 2H), 1.80–1.71 (m, 2H), 1.41–1.36 (m, 4H), 0.93 (t, *J* = 6.2 Hz, 3H). ^13^C NMR (126 MHz, CDCl_3_) δ 190.93, 159.21, 157.17, 151.60, 141.14, 134.21, 130.95, 129.49, 129.17, 125.17, 124.18, 122.32, 115.61, 115.08, 68.33, 31.61, 29.25, 25.65, 22.60, 14.06. Elemental analyses: Found (calculated): C, 76.21 (76.22); H, 7.40 (7.41); N, 4.65 (4.68).

**N-4-fluorobenzylidene-4-(octyloxy)benzenamine, I*8***

Yield: 93.0%; mp 88.0 °C, FT-IR (ύ, cm^−1^): 2935, 2862 (CH_2_ stretching), 1632 (C=N), 1550 (NO_2_), 1362 (NO_2_). ^1^H NMR (500 MHz, CDCl_3_) δ 8.66 (s, 1H, CH=N), 7.89 (d, 1H), 7.86 (d, 1H), 7.52 (d, 1H), 7.43 (dd, 1H), 7.27 (d, 1H), 7.25–7.22 (m, 1H), 7.03–6.99 (m, 2H), 4.05 (t, 2H, OCH_2_), 1.78–1.71 (m, 2H, CH_2_), 1.43–1.30 (m, 10H, 5 CH_2_), 0.89 (t, 3H, CH_3_). Elemental analyses: Found (calculated): C, 77.03 (76.89); H, 8.00 (7.92); N, 4.28 (4.18).

**N-4-fluorobenzylidene-4-(hexadecyloxy)benzenamine, I*16***

Yield: 95.0%; mp 79.0 °C, FT-IR (ύ, cm^−1^): 2932, 2854 (CH_2_ stretching), 1625 (C=N), 1555 (NO_2_), 1365 (NO_2_). ^1^H NMR (500 MHz, CDCl_3_) δ 8.65 (s, 1H, CH=N), 7.90 (d, 1H), 7.83 (d, 1H), 7.55 (d, 1H), 7.45 (m, 1H), 7.29 (d, 1H), 7.20–7.15 (m, 1H), 7.03–6.80 (m, 2H), 4.06 (t, 2H, OCH_2_),1.77–1.70 (m, 2H, CH_2_), 1.43–1.31 (m, 26H, 13 CH_2_), 0.91 (t, 3H, CH_3_). Elemental analyses: Found (calculated): C, 79.22 (79.21); H, 9.63 (9.47); N, 3.19 (3.25).

**N-(4-fluoro-3-nitrobenzylidene)-4-(hexyloxy)aniline, II*6***

Yield: 95.1%; mp 98.0 °C, FT-IR (ύ, cm^−1^): 2928, 2845 (CH_2_ stretching), 1610 (C=N), 1555 (NO_2_), 1360 (NO_2_). ^1^H NMR (500 MHz, CDCl_3_) δ 8.55 (t, *J* = 7.1 Hz, 1H, Ar-H), 8.49 (m, 1H, Ar-H), 8.19 (s, 1H, CH=N), 7.38 (m, 1H, Ar-H), 7.26 (m, 1H, Ar-H), 6.93 (t, *J* = 8.3 Hz, 1H, Ar-H), 4.04–3.91 (m, 2H, OCH_2_), 1.79 (d, *J* = 5.8 Hz, 2H, CH_2_), 1.47 (s, 2H, CH_2_), 1.38–1.29 (m, 4H, 2CH_2_), 0.94–0.88 (m, 3H, CH_3_). ^13^C NMR (126 MHz, CDCl_3_) δ 188.87 (C), 158.81 (C), 156.68 (d, *^1^J_C_*_,*F*_ = 269.2 Hz, CF), 153.20 (CH) 134.50 (d, ^2^*J*_C,F_ = 24.1 Hz, CH), 127.38 (d, ^2^*J*_C,F_ = 29.5 Hz, C), 119.05 (d, ^3^*J*_C,F_ = 21.6 Hz, CH), 126.07 (CH), 122.56 (CH), 122.28 (C), 115.18 (CH), 68.11 (OCH_2_), 31.51 (CH_2_), 29.18 (CH_2_), 25.83 (CH_2_), 22.51 (CH_2_), 14.01 (CH_3_). Elemental analyses: Found (calculated): C, 66.24 (66.26); H, 6.14 (6.15); N, 8.10 (8.13).

**N-(4-fluoro-3-nitrobenzylidene)-4-(octyloxy)aniline, II*8***

Yield: 93.7%; mp 89.0 °C, FT-IR (ύ, cm^−1^): 2930, 2846 (CH_2_ stretching), 1612 (C=N), 1552 (NO_2_), 1360 (NO_2_). ^1^H NMR (500 MHz, CDCl_3_) δ 8.70 (s, 1H, CH=N), 8.49 (m, 1H, Ar-H), 8.20 (m, 1H, Ar-H), 7.68 (m, 1H, Ar-H), 7.23 (m, 2H, Ar-H), 6.98 (m, 2H, Ar-H), 4.04–3.92 (m, 2H, OCH_2_), 1.75 (m, 2H, CH_2_), 1.44–1.30 (m, 10H, 5CH_2_), 0.96–0.88 (m, 3H, CH_3_). Elemental analyses: Found (calculated): C, 67.72 (67.56); H, 6.77 (6.65); N, 7.52 (7.43).

**N-(4-fluoro-3-nitrobenzylidene)-4-(hexadecyloxy)aniline, II*16***

Yield: 95.8%; mp 75.0 °C, FT-IR (ύ, cm^−1^): 2925, 2848 (CH_2_ stretching), 1615 (C=N), 1550 (NO_2_), 1362 (NO_2_). ^1^H NMR (500 MHz, CDCl_3_) δ 9.85 (s, 1H, CH=N), 8.50 (s, 1H, Ar-H), 8.23–8.15 (m, 1H, Ar-H), 7.39 (t, *J* = 9.4 Hz, 1H, Ar-H), 7.30–7.16 (m, 2H, Ar-H associated with the solvent peak), 6.96 (m, 2H, Ar-H). 4.01–3.95 (m, *J* = 7.3, 5.4 Hz, 2H, OCH_2_), 1.85–1.75 (m, *J* = 16.7, 9.9 Hz, 2H, CH_2_), 1.51–1.42 (m, J = 5.2 Hz, 2H, CH_2_), 1.39–1.20 (m, *J* = 47.2 Hz, 24H, 12 CH_2_), 0.88 (t, *J* = 6.5 Hz, 3H, CH_3_). ^13^C NMR (126 MHz, CDCl_3_) δ 188.87, 158.81, 153.18, 134.62, 133.82, 132.04, 127.49, 127.25, 126.07, 122.56, 122.25, 119.13, 116.54, 115.86, 115.74, 115.18, 68.42 (OCH_2_), 32.04 (CH_2_), 29.81 (CH_2_), 29.77 (CH_2_), 29.71 (CH_2_), 29.68 (CH_2_), 29.52 (CH_2_), 29.48 (CH_2_), 29.37 (CH_2_), 29.31 (CH_2_), 26.15 (CH_2_), 22.81 (CH_2_), 14.25 (CH_3_). Elemental analyses: Found (calculated): C, 71.86 (71.87); H, 8.50 (8.53); N, 5.75 (5.78).

## 4. Conclusions

The new two-rings azomethine series, *N-4-florobenzylidene-4-(alkoxy)benzenamine* (**I***n*) and *N-(4-fluoro-3-nitrobenzylidene)-4-(alkyloxy)aniline* **(II***n***)** were synthesized and characterized by experimental and theoretical approaches. The molecular structures were elucidated via elemental analyses, FT-IR, and NMR spectroscopy. Experimental characterizations of their mesomorphic behaviors were conducted by DSC and POM. The theoretical simulation of geometrical structure investigations were carried out by the DFT calculation method.

The study revealed that:

1. All compounds of the synthesized laterally neat group (**I***n*) were mesomorphic, exhibiting liquid crystalline mesophases monotropically.
2. The observed smectic A phase in group **I***n* covered all lengths of terminal alkoxy chains.
3. The compounds in group **II***n* were non-mesomorphic, which attributed to the steric hindrance of the large size of the nitro group, which disrupted the SmA phase.
4. The molecular geometries of the investigated compounds were highly impacted by the electronic nature, location, and size of the attached NO_2_ substituent.
5. The high dipole moment and steric hindrance of the NO_2_ moiety explain their non-mesomorphic properties.
6. The electronic and geometric structures of the nitro group through intermolecular interactions have an essential role that affects the thermal and physical properties of the present investigated compounds.

## Figures and Tables

**Table 1:** Phase transition temperatures (°C), enthalpy of transition ΔH, kJ/mole, and mesophase temperature range, ΔT, for groups N-4-florobenzylidene-4-(alkoxy)benzenamine (**I***n*) and N-(4-fluoro-3-nitrobenzylidene)-4-(alkyloxy)aniline (**II***n*)**.**

Cycle	Upon Heating		Upon Cooling				
Comp.	*T*_Cr-I_	Δ*H*_Cr-I_	*T*_I__-__SmA_	Δ*H*_I-SmA_	*T*_SmA-Cr_	Δ*H*_SmA-Cr_	Δ*T*
**I** *6*	84.2	35.64	66.9	2.15	61.5	27.6	5.4
**I** *8*	88.2	39.35	71.0	2.05	54.7	32.4	16.3
**I** *16*	79.1	36.72	76.3	1.98	<20.0	-	>56.3
**II** *6*	98.2	40.74	-	-	-	-	-
**II** *8*	88.7	38.27	-	-	-	-	-
**II** *16*	74.8	50.61	-	-	-	-	-

Cr-to-isotropic liquid phase (Cr-I) denotes transition from solid to the isotropic mesophase; I-smectic A (I-SmA) denotes transition from isotropic mesophase to the SmA mesophase; SmA-Cr denotes transition from SmA to the solid phase.

**Table 2:** DFT calculated thermal parameters using B3LYP/6-311G** method.

Compound	ZPE (Kcal/Mol)	Thermal Energy (Kcal/Mol)	Enthalpy (Kcal/Mol)	Gibbs Free Energy (Kcal/Mol)	Entropy (Cal mol.k)	Total Energy (Hartree)
**I** *6*	232.110	245.514	246.107	198.082	161.077	−966.673
**II** *6*	233.080	248.147	248.739	196.560	175.010	−1171.136
**I** *8*	268.005	283.060	283.652	231.671	174.347	−1045.237
**II** *8*	268.850	285.632	286.224	229.633	189.806	−1249.700
**I** *16*	411.206	433.130	433.723	364.161	233.314	−1359.495
**II** *16*	412.130	435.744	436.336	362.232	248.547	−1563.957

**Table 3:** E_HOMO_, E_LUMO_, ∆E, the dipole moment, and the polarizability calculated using B3LYP/6-311G** method for the present groups **I***n* and **II***n*.

Compound	E_HOMO_ (ev)	E_luMO_ (ev)	∆E (ev)	IE (ev)	EA (ev)	Dipole Moment (Debye)	Polarizability (Bohr^3^)
**I** *6*	−5.820	−1.871	3.949	5.820	1.871	3.4343	253.23
**II** *6*	−6.185	−3.207	2.978	6.185	3.207	8.3129	277.41
**I** *8*	−5.826	−1.878	3.948	5.826	1.878	3.2523	275.53
**II** *8*	−6.178	−3.224	2.954	6.178	3.224	7.2100	299.49
**I** *16*	−5.822	−1.872	3.950	5.822	1.872	3.5361	372.25
**II** *16*	−6.177	−3.207	2.970	6.177	3.207	8.3918	396.18

## Supplementary Materials

The following are available online. Figure S1: DSC thermograms of **I**6 derivative upon second heating/cooling scan with a rate of 10 °C/min. Figure S2: DSC thermograms of **I**16 derivative upon second heating/cooling scan with a rate of 10 °C/min. Figure S3: DSC thermograms of **II***6* derivative upon second heating/cooling scan with a rate of 10 °C/min. Figure S4: DSC thermograms of **II***8* derivative upon second heating/cooling scan with a rate of 10 °C/min.

## Sample Availability

Samples of the compounds **I***n*, **II***n* are available from the authors.
