# Supplementary material for: New Nitro-Laterally Substituted Azomethine Derivatives; Synthesis, Mesomorphic and Computational Characterizations
Source: Molecules. 2021 Mar 30;26(7):1927. doi: 10.3390/molecules26071927 (PMC8036588; doi:10.3390/molecules26071927)
Supplement: Supplementary file 1 [file molecules-26-01927-s001.pdf]

Supplementary Materials

New Nitro-Laterally Substituted Azomethine Derivatives; Synthesis, Mesomorphic and Computational Characterizations

Mohamed A. El-atawy [1,2,*], Magdi M. Naoum [3], Salma A. Al-Zahrani [4] and Hoda A. Ahmed [3*]

[1] Chemistry Department, Faculty of Science, Alexandria University, P.O. 426 Ibrahemia, Alexandria, 21321, Egypt
[2] Chemistry Department, Faculty of Science, Taibah University, Yanbu, 46423, Saudi Arabia
[3] Department of Chemistry, Faculty of Science, Cairo University, Cairo 12613, Egypt, magdinaoum@yahoo.co.uk
[4] Department of Chemistry, College of Sciences, University of Ha'il, Ha'il 2440, Saudi Arabia, s.alzahrane@uoh.edu.sa
* Correspondence: mohamed.elatawi@alexu.edu.eg (M.A.E.); ahoda@sci.cu.edu.eg (H.A.A.)

1. Materials

4-hexyloxyaniline, 4-(octyloxy)aniline, 4-(hexadecyloxy)aniline, 4-fluorobenzaldehyde, nitric acid, sulfuric acid and ethanol were purchased from Sigma Aldrich (Germany).

2. Synthesis of N-4-florobenzylidene-4-(alkoxy)benzenamine (In)

Ethanolic solution of 4-fluorobenzaldehyde (10 mmol) was added to a solution of the appropriate alkoxy aniline namely (4-hexyloxyaniline, 4-(octyloxy)aniline or 4-(hexadecyloxy)aniline,). Then the mixture was refluxed for two hours. The reaction mixture was allowed to cool to room temperature and the perciptated solid was filtered. The obtained product was recrystallized from ethanol.

3. Synthesis of 4-fluoro-3-nitrobenzaldehyde

4-Fluorobenzaldehyde (10 mmol) was dissolved in 5 mL of conc. sulfuric acid, and the mixture cooled to 0 °C then a mixture of nitric acid (1.2 equiv.) and 1 mL of conc. sulfuric acid was added slowly to the reaction mixture. The reaction mixture was allowed to warm gradually to room temperature and then further stirred for 30 minutes at room temperature. The mixture was poured into 50 mL of ice-cold water, filtered and washed with cold water.

4. Synthesis of N-(4-fluoro-3-nitrobenzylidene)-4-(alkyloxy)aniline (IIn)

Ethanolic solution of 4-fluoro-3-nitrobenzaldehyde (10 mmol) was added to a solution of the appropriate alkoxy aniline namely (n = 6, 8, and 16). The mixture was stirred for three hours at room temperature. The reaction mixture was cooled to 0 °C and the formed solid was filtered. The obtained product was recrystallized from ethanol.

5. Characterization

Melting points were determined by MEL-TEMP II melting point apparatus in open glass capillaries and were uncorrected. The IR spectra were recorded as potassium bromide (KBr) discs on a Perkin-Elemer FT-IR (Fourier-Transform Infrared Spectroscopy), college of Science, Taibah University. The NMR spectra were carried out at ambient temperature (~25 °C) on a (JEOL) 500 MHz spectrophotometer using tetra methyl silane (TMS) as an internal standard, NMR Unit, Faculty of Science, Mansoura University. Chemical shift was recorded as δ values in parts per million (ppm), and the signals were reported as s (singlet), d (doublet), t (triplet) and m (multiplet). Elemental analyses were analyzed at the Micro analytical Unit, Faculty of Science, Cairo University.

TA Instruments Co. (Q20 Differential Scanning Calorimeter, DSC; USA) was used for recording phase transitions. DSC calibration was carried using lead and indium

melting temperature and enthalpy. Samples of 2–3 mg were used in aluminum pans for DSC investigation. The heating rate was 10°C/min in nitrogen gas as an inert atmosphere (30 ml/min). All transitions measured were for the second heating scan.

Transition temperatures for the prepared compounds were checked and phases identified by Polarized optical microscope (POM, Wild, Germany) attached with Mettler FP82HT hot stage.

6. Computational Method

All DFT calculations were performed using the Gaussian 09 software. A conformational search for obtaining the most stable conformer was done using the semi-empirical PM3 method. The most stable conformer was subjected to full geometrical optimizations using the DFT and Becke's three-parameter hybrid exchange functional in combination with the gradient-corrected correlation functional of Lee, Yang and Parr B3LYP/6-311G** method without any constraints to calculations. All calculated structures were found to be true minima, i.e., no imaginary frequencies were observed after the ground state geometry optimization.

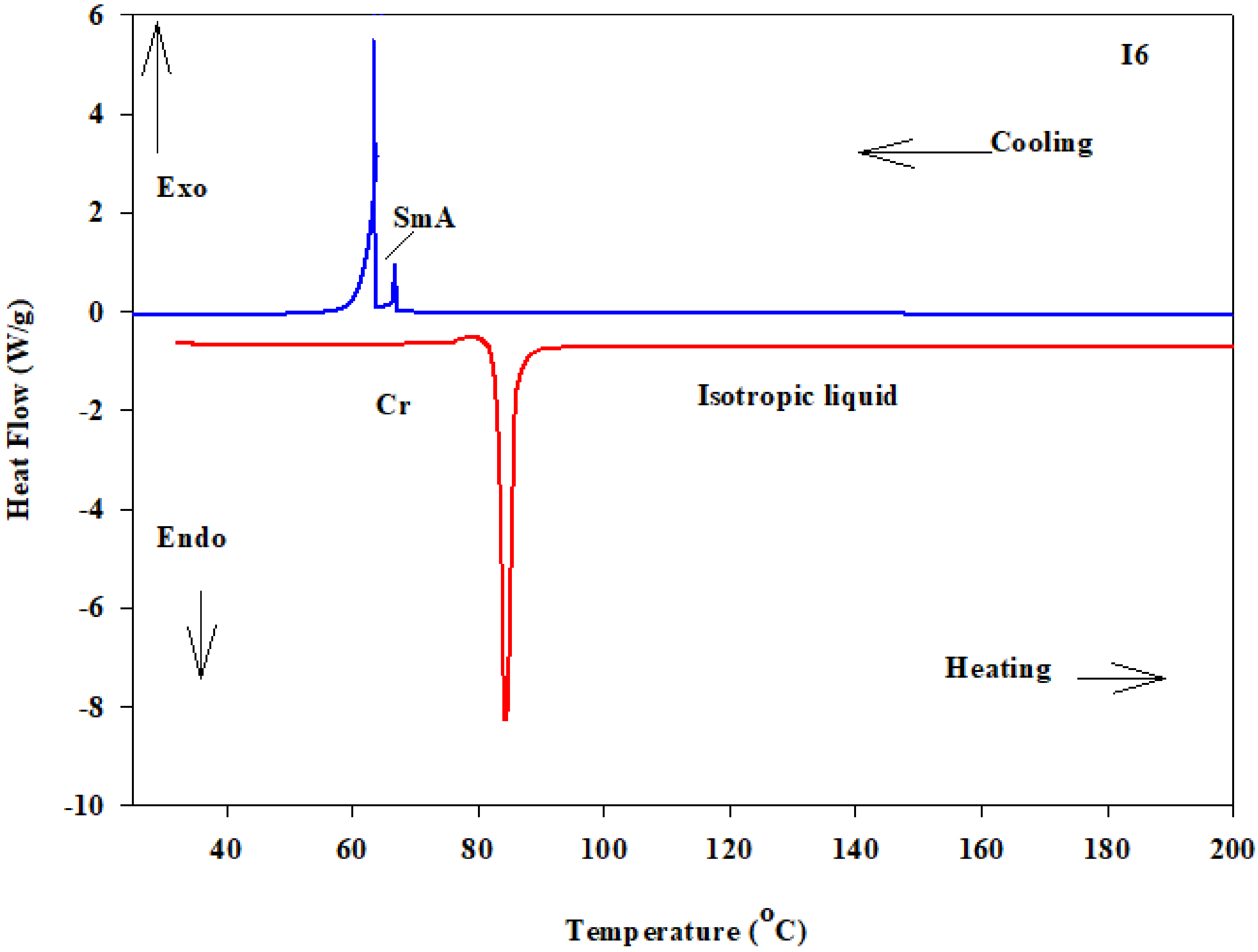

Figure S1. DSC thermograms of **I6** derivative upon second heating/cooling scan with rate 10 °C /min.

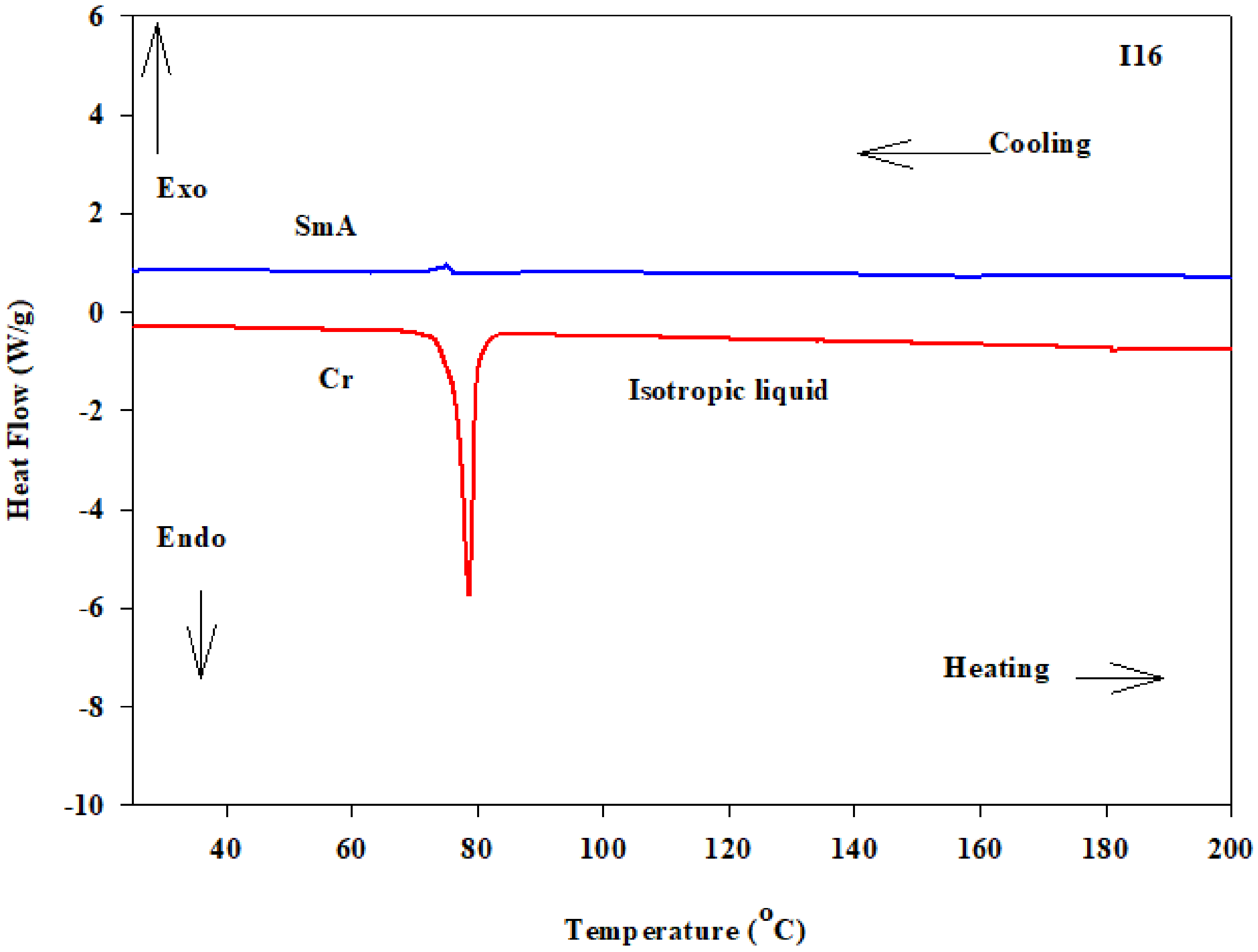

Figure S2. DSC thermograms of **I16** derivative upon second heating/cooling scan with rate 10 °C /min.

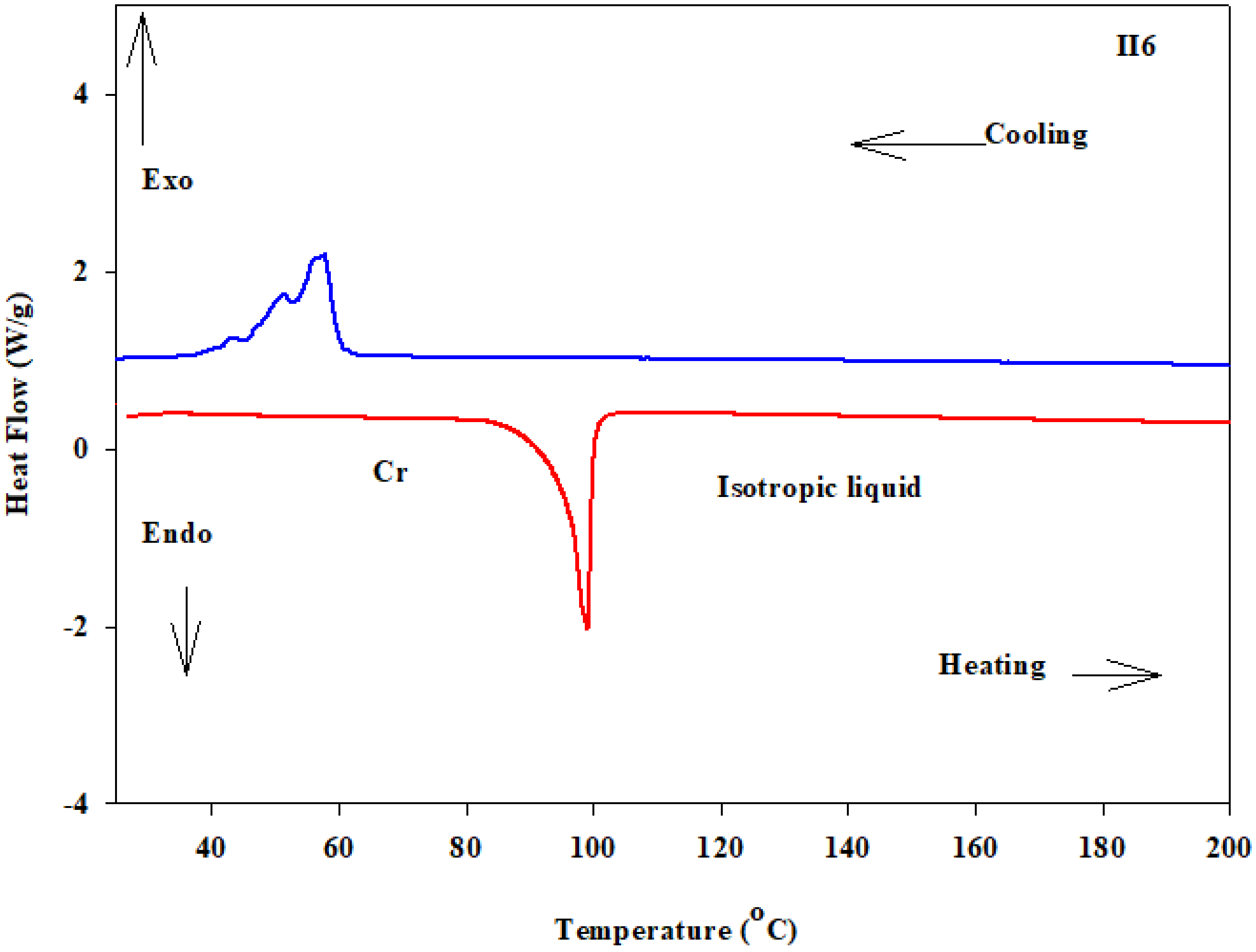

Figure S3. DSC thermograms of **II6** derivative upon second heating/cooling scan with rate 10 °C /min.

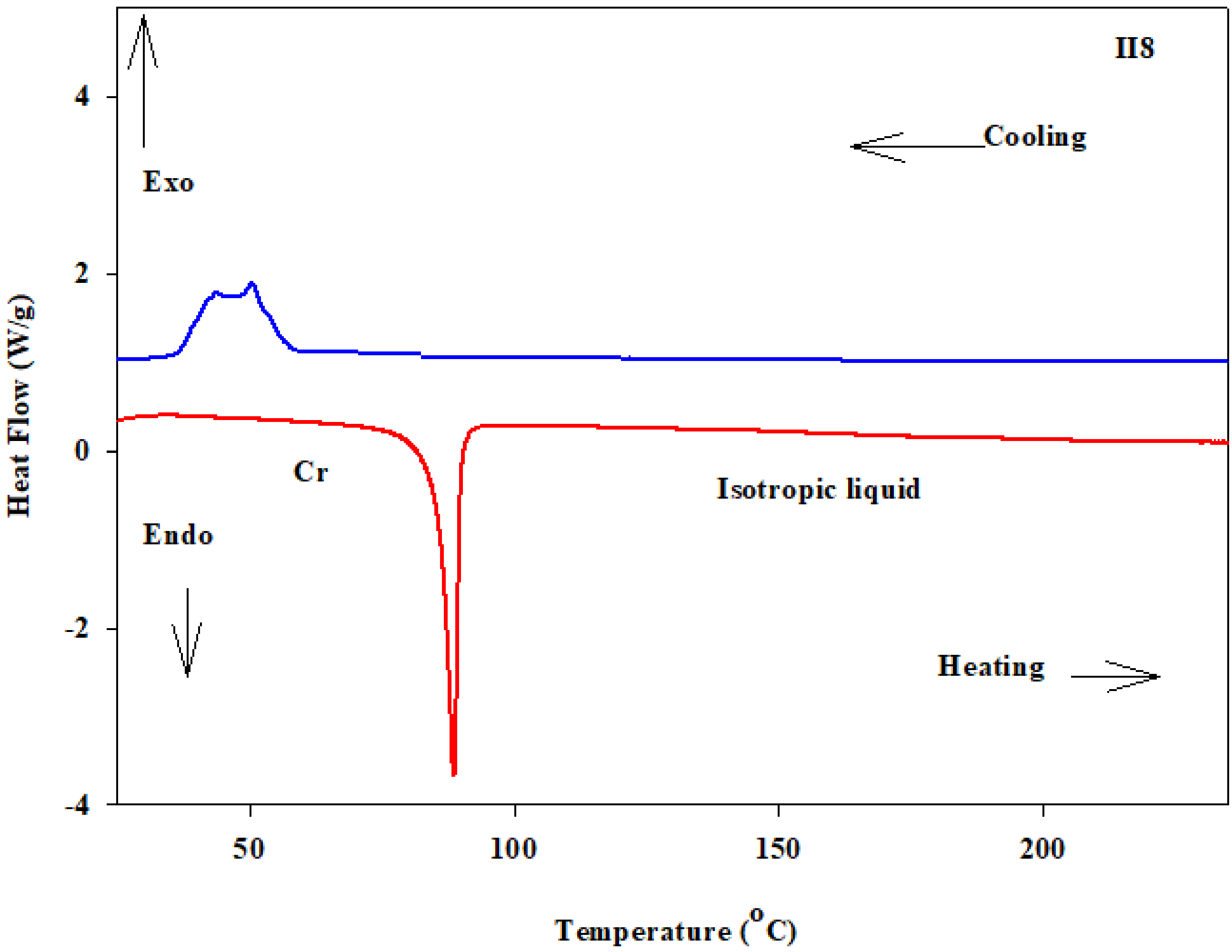

Figure S4. DSC thermograms of **II8** derivative upon second heating/cooling scan with rate 10 °C /min.
